# Conflict exposure and mental health: a survey of adolescent girls and young women in Myanmar post the 2021 coup d’état

**DOI:** 10.1186/s13031-025-00668-y

**Published:** 2025-05-16

**Authors:** Isabelle Pearson, Elaine Chase, Cing Van Kim, Nang Ma San, Hkawn Ja, Zin Mar Hlaing, Nandar Oo, Khin Lae, Ei Ei Soe, Brooke Zobrist, Cathy Zimmerman, Meghna Ranganathan

**Affiliations:** 1 Department of Global Health and Development, London School of Hygiene and Tropical Medicine, 15-17 Tavistock Pl, London, WC1H 9SH UK; 2 Institute of Education, University College London, London, UK; 3 Girl Determined, Yangon, Myanmar

**Keywords:** Conflict, Mental Health, Adolescents, Children, Gender, Wellbeing

## Abstract

**Background:**

Following the 2021 military coup in Myanmar, adolescent girls and young women have faced a multitude of threats to their health and wellbeing. Beyond direct exposure to armed combat, injuries and loss of life, they are also experiencing displacement, family separation, and restricted access to education and healthcare. These challenges are further compounded by military-imposed restrictions on humanitarian aid and access to the country. This study sought to address a critical gap in understanding how exposure to conflict in Myanmar is impacting adolescent girls’ mental health.

**Methods:**

We conducted a survey, co-developed with peer-researchers, with 750 girls and young women aged 10 to 21 years from disproportionately disadvantaged communities across Myanmar. The survey included questions on participants’ demographics, their exposure to conflict-related stressors and their self-reported depressive symptoms.

**Results:**

Participants reported widespread exposure to traumatic conflict-related stressors and high levels of depressive symptoms. There was a significant positive association between the number of different conflict-related stressors experienced by the study participants and the number of depressive symptoms reported. Additionally, participants who were living away from their parents reported significantly higher levels of depressive symptoms, emphasising the psychological implications of family separation during conflict.

**Conclusions:**

Our results highlight the urgent need for tailored interventions for adolescents, particularly girls and young women, who are exposed to armed conflict. In contexts of protracted instability and uncertainty, such as in Myanmar, interventions should explore ways to foster a sense of social safety, especially among adolescent girls who are displaced or living away from their families. Strengthening social safety systems may help to reduce the adverse mental health impacts of conflict-related stressors.

## Introduction

Globally, there are 1.2 billion adolescents, 90% of whom live in low- and middle-income countries and 10% of whom live within armed conflict settings [1]. The direct and indirect effects of war and instability can have severe impacts on young people’s short and longer-term mental and physical health and wellbeing [2, 3]. Conflict directly impacts physical health through increased risk of death, injury and disability, and directly impacts mental health through psychological trauma, with one in five people in conflict settings experiencing symptoms of depression, anxiety, post-traumatic stress disorder (PTSD), bipolar disorder or schizophrenia [4]. Indirect effects of conflict include, for example, the loss of essential services such as healthcare and education, which are disrupted or even targeted [5]. When these critical service gaps co-occur with conflict-associated stressors, including forced displacement, increased disease prevalence and rising poverty levels, the impacts on adolescents’ psychosocial wellbeing can be severe [3, 6].

Since the 2021 military coup, which overthrew the ruling democratic party and exacerbated nationwide political instability, adolescents in Myanmar have faced a complex nexus of individual and societal stressors impacting their physical and mental health and wellbeing [7]. These events further compounded the deep socio-economic effects of the COVID-19 pandemic, and military-enforced national conscription laws have further threatened the safety and personal freedoms of young people across the country [8, 9]. While adolescents and youth suffered serious effects of the coup, many also emerged as prominent figures in the resistance, particularly through their participation in the civil-disobedience movement, which included long-term strikes from schools and workplaces [5]. In 2024, Myanmar was ranked second in the world on the Armed Conflict Location and Event Data’s conflict severity index, second only to Palestine [10]. Despite this, our understanding of adolescent mental health in Myanmar remains limited, as does the critical knowledge needed to inform targeted interventions that safeguard the mental health and wellbeing of young people.

During conflict, multiple co-stressors—such as family separation and unmet humanitarian needs for shelter, food and healthcare—can increase anxiety and depression among adolescents [11, 12]. At the same time, evidence highlights social support as a key protective factor for adolescent mental health [13]. Yet, during conflict and civil unrest, young people’s access to protective social networks can become severely restricted due to forced displacement and the erosion of education and community-based social support systems [13–15]. The implications of these conflict-related co-stressors on adolescent mental health have been documented across various conflicts. For example, a global systematic review including 7,920 war-exposed children and adolescents aged 5–17 years identified prevalence rates of depression as high as 43%, PTSD at 47% and anxiety at 27%, significantly higher than the general population [2]. Similarly, a systematic review on conflict-exposed children and adolescents from the Middle East found that exposure to war-related traumatic experiences was correlated with an increased prevalence of mental health problems [6]. Among children and adolescents living in the Gaza Strip, for example, PTSD was estimated as high as 53.5% [16].

Conflict-related risks to adolescents’ physical and mental health are compounded by existing inequalities related to a young persons’ gender, sexual orientation, ethnicity, disability, caste and religion [17]. Adolescent girls and young women in particular face unique risks during conflict, including increased sexual and gender-based violence, early and forced marriage and reduced sexual and reproductive health services [18, 19]. Furthermore, family efforts to protect girls and young women from harm during conflict, such as early marriage and highly restricted mobility, can compound the conflicts’ impact on their mental health by limiting girls’ access to social support networks, peer relationships, and educational opportunities, particularly compared to their male peers [20]. While depression and anxiety are generally more common among adolescent girls even outside of conflict settings, exposure to conflict may intensify these gender disparities by reinforcing harmful gendered norms and limiting resources for coping and support [21, 22]. The effects of these risks on girls’ mental health have been shown through studies of various conflicts. For example, studies on PTSD among school students in Syria have found that PTSD symptoms were significantly higher among girls than boys [23, 24]. Among Palestinian refugees aged 11–16 years attending schools in the Gaza Strip, West bank, and United Nations camps across the region, girls more frequently reported feeling lonely and worried, whereas boys were more likely to miss school [25]. Likewise, Betancourt et al. [26] showed that among child soldiers in Sierra Leone, girls reported higher rates of depression and anxiety than boys.

In Myanmar, there is a dearth of evidence on how the conflict is affecting adolescent mental health specifically, particularly among young women. A 2021 study of 7,720 adults predicted high rates of anxiety (58%) and depression (61%) [27]. Similarly, a telephone survey of 1,038 adults found that one third reported a probable mental health concern, with 14.3% reporting likely depression and 22.2% reporting likely anxiety in the months following the 2021 coup [28]. However, these concerning figures do not capture the potentially disproportionate influence of the conflict on adolescent girls and young women.

Although almost one in five adolescent girls currently live in conflict zones, our understanding of how interacting risks affect adolescent girls’ mental health remains limited, and filling these gaps in our knowledge is essential to address the unique risks faced by these populations [29]. In Myanmar, the protracted conflict reinforces entrenched gender-inequalities, which disproportionately affect adolescent girls and young women [30]. Yet, there has been little evidence about the frequency and severity of conflict exposure among girls and young women, and how these exposures may be impacting their mental health.

### Aim and objectives

This study aims to analyse associations between conflict-related stressors and the mental health of adolescent girls and young women in Myanmar. Specifically, the objectives are to:

1. Examine the prevalence of exposure to conflict-related stressors among adolescent girls and young women;
2. Measure the prevalence of depressive symptoms; and.
3. Examine the association between the severity of conflict exposure and reported depressive symptoms.

## Methods

### Study design and participants

We conducted this cross-sectional study in collaboration with Myanmar-based NGO, Girl Determined (GD). The survey was co-developed with six peer-researchers from GD (EES, HJ, KL, NDO, NMS, ZMH). The peer-researchers received training on data collection methods and research ethics from IP and CVK to each conduct 125 structured surveys. Five sites were chosen for data collection: Mandalay Region, Yangon Region, Kachin State, Shan State and Tanintharyi Region. These sites were selected because they are the home states of the peer-researchers, thus safely accessible during the ongoing conflict. Moreover, these are locations where GD implement their empowerment, digital literacy and sports programmes. Within each region, we randomly selected sites where GD’s empowerment programme, “Circles”, was ongoing. We selected sufficient sites for each peer-researcher to conduct 125 surveys, whilst purposely excluding any selected sites that were unsafe due to conflict. Two researchers were based in Mandalay Region, one in Kachin State, one in Shan state, one in Yangon Region and one researcher conducted her surveys at sites across both Yangon and Tanintharyi Regions. At each site, we invited every girl registered with GD (most of whom are aged 10–18 years) to complete a survey and the main reason girls declined to take part in the study was school exams. Every participant was enrolled in GD’s “Circles” programme at the time of survey. “Circles” covers topics such as self-confidence, assertiveness, ethical and religious discrimination, trafficking and safe migration practices, stress management, relationships (both romantic and friendships), drug awareness, goal-setting and sexual and reproductive health education [31]. GD prioritises reaching marginalised girls through settings such as camps for internally displaced people (IDPs), Buddhist monastic schools, and dormitories for girls from rural areas. Registration is not based on individual-level criteria and instead occurs at the group level. For example, all girls aged 10–18 within specific IDP camps or boarding houses serving low-income rural families are eligible to participate. Therefore, all girls enrolled in “Circles” generally represent the lowest socio-economic quintiles in Myanmar, and our study sample includes disproportionately high numbers of girls living away from their families due to GD’s targeting strategy rather than reflecting broader national adolescent living arrangements. Additionally, high school enrolment in our sample is likely also reflective of GD’s recruitment strategy, which targets sites offering educational services.

### Procedures

The survey tool was co-developed with the peer-research team in September 2023 in Yangon. Most survey questions are based on existing tools, which, where possible, had been validated for use with adolescents in low-income settings in South-East Asia. Due to the scarcity of research tools validated with adolescents in Myanmar, and time and resource constraints, formal validation in our setting was not feasible. However, the research team, including local peer-researchers very familiar with the study population, carefully reviewed the entire survey to identify and revise potentially confusing or culturally inappropriate language, ensuring that phrasing was clear, simple and contextually relevant. The tool was translated by CVK, and translation accuracy was confirmed by bilingual members of GD staff. We further revised the tool through validation sessions with the peer-researcher team following the thorough piloting exercises conducted in Yangon in September 2023. During these validation sessions, we discussed the interpretation of questions during the pilots and amended any unclear or complicated phrasing.

The survey was conducted between September 2023 and January 2024. The survey measures relevant for this study are outlined below. For all included Likert scales, a card was presented to participants with a visual representation of the scale using smiling faces and a green to red colour code. All data was collected using Kobo Toolbox on mobile phones.

### Ethics and consent

We collected informed assent and either parental consent or the consent of a trusted adult from each participant included in this study. Special safeguarding and security measures were in place for this study, to ensure the safety of all participants and field teams, including service referral processes and staff security check-ins. All surveys were conducted in enclosed rooms where privacy could be maintained. Before enquiring about sensitive topics, participants were reminded of the option to skip any questions. Each survey took approximately one hour, and breaks were offered where needed. Participation was voluntary, no monetary compensation was provided as the surveys were conducted as part of participants’ ongoing GD programme schedule and alternative activities were provided for participants who did not want to take part in the survey. Ethical approval for this research was received from the London School of Hygiene and Tropical Medicine Ethics Committee (ref 28458-2) and a local research ethics committee in Myanmar.

### Measures

#### Demographics

We collected demographic data on participants’ age, religion, ethnicity, highest school grade and their current education enrolment status.

#### Living situation

Participants’ living situations were assessed based on questions about where they were currently living, who they lived with and their primary caregiver.

#### Exposure to conflict-related stressors

To measure the participants exposure to conflict-related stressors, we adapted four questions (see 4–7 below) from the Child War and Trauma Questionnaire (Child subset) [32] and added three (see 1–3 below) that were more suited to capture our participants’ situations, these questions were developed from discussions with local experts. After an introduction about the ongoing political conflict, we asked participants: “Thinking about between now and 2021, please answer yes or no to the following questions: 1) have you had to flee your home or move house because of the conflict, even if only for one night?; 2) have you had to move schools due to the conflict?; 3) have you been prevented from having access to your usual education due to the conflict?; 4) have you been separated from your parents/caregivers due to conflict?; 5) have you lost any family members due to the conflict?; 6) have you been directly exposed to armed combat (such as shelling, shooting, bomb explosion)?; 7) have you suffered any injury as a result of the conflict?”. Based on affirmative responses, a score for exposure to conflict-related stressors was calculated, which could range from zero to seven. Responses were summed to create a cumulative conflict exposure score (0–7). For analytical purposes and consistent with prior literature [33], respondents were grouped into categories: no stressors, 1–2 stressors, and 3 or more stressors.

#### Mental health

Mental health was assessed using a proxy depressive symptoms measurement tool from the Global Early Adolescent Health Survey (GEAS) [34]. This question set contained six questions, to which participants responded on a 5-point Likert scale of “Agree a lot” to “Disagree a lot”. Respondents also had the option to refuse any question. The six question were: “1) In general, I see myself as a happy person; 2) I blame myself when things go wrong; 3) I worry for no good reason; 4) I am so unhappy, I cannot sleep at night; 5) I feel sad; 6) I am so unhappy I think of harming myself”. Following GEAS guidelines, we calculated a summative score based on negative responses to questions 2–6, and affirmative responses to question 1. Depressive symptom scores could range from zero (no depressive symptoms) to six (all six depressive symptoms present). The Cronbach’s alpha score was initially < 0.6, so we removed question one from the question set which resulted in a Cronbach’s alpha of 0.62. Therefore, for our analyses only questions 2–5 were used to calculate the depressive symptom score, ranging from zero to five.

### Statistical analyses

All statistical analyses included only participants who fully answered the relevant question sets. All hypotheses were defined a priori and based on our previous qualitative research findings [30]. Descriptive statistics were used to summarise participant demographics. Participants were characterised into two developmental age groups: early adolescents (10–14 years) and mid-late adolescents (15–21 years), informed by distinctions described by Sawyer et al. [35]. Differences between these age groups in demographic variables and exposure to conflict-related stressors were assessed using Chi-square tests for categorical variables, independent t-tests for continuous variables with normal distribution, and Spearman’s rank correlation for non-normally distributed or count-based data.

Conflict-related exposure variables were analysed both individually and cumulatively. Cumulative conflict exposure was analysed categorically (0, 1–2, ≥ 3 stressors) to simplify interpretation and maintain sufficient statistical power. The association between the number of conflict-related stressors and the number of depressive symptoms was examined using Spearman’s rank correlation due to the count-based and non-normal nature of these variables. Ordinal logistic regression was used to analyse the associations between exposure to conflict-related stressors (both grouped and individually) and the number of depressive symptoms. Adjusted models controlled for potential confounders, specifically age and whether respondents lived with their birth parents to explore potential effect modification. It is important to note that one of seven conflict-related stressors assessed (being separated from parents/caregivers due to the conflict) may overlap conceptually with the stratification variable of living away from parents. Therefore, participants in the ‘living away’ subgroup may have more frequently endorsed this exposure item, which could constrain variation in exposure counts within that subgroup and limit our ability to detect associations at lower exposure levels.

Descriptive statistical analyses were conducted during co-production analysis and data interpretation workshops with the peer-researchers using Microsoft Excel and all regression analyses were conducted by IP in R Studio (version 4.3.0).

## Results

### Demographics

Table 1 presents the main demographics of our study sample. Study participants included 750 adolescent girls and young women, aged 10–21 years. The median age for all participants was 14 years; 13 years for the early adolescent group and 16 years for the mid-to-late adolescent group. Participants were living in villages and peri-urban areas primarily in the Mandalay (33.3%) and Yangon Regions (26.8%), plus Kachin State (16.7%), Shan State (16.7%) and Tanintharyi Region (6.5%).

Notably, half of the participants (50.8%) were living away from their parents at the time of the survey. Family separation was more common among middle or older adolescents (65.0%) than early adolescents (42.2%). Those living away from their parents were most often living at nunneries, boarding houses, or with other family members. Overall, 97.5% of our participants were enrolled in some form of education at the time of the survey. Bamar was the main ethnicity in both Yangon (46.8%) and Mandalay Regions (72.8%), Kachin was the main ethnicity in Kachin State (94.4%), Pa’O in Shan State (72.0%) and Karen in Tanintharyi Region (95.9%).

**Table 1:** Participant demographics (*n* = 750)

	Early Adolescents: 10–14 years (*n* = 467)	Mid-Late Adolescents: 15–21 years (*n* = 283)	Total (*n* = 750)
Education			
Enrolled in school	464 (99.4%)	267 (94.3%)	731 (97.5%)
Primary (G1–G4)	121 (25.9%)	2 (0.7%)	123 (16.4%)
Secondary (G5–G11)	343 (73.4%)	276 (97.5%)	619 (82.5%)
Other/Refused	3 (0.6%)	5 (1.8%)	8 (1.1%)
Living Situation			
Live with parents	270 (57.8%)	99 (35.0%)	369 (49.2%)
Live away from parents	197 (42.2%)	184 (65.0%)	381 (50.8%)
Nunnery	146 (31.3%)	146 (51.6)	292 (38.9%)
Boarding house/school	11 (2.4%)	17 (6.0%)	28 (3.7%)
With other family members	23 (4.9%)	10 (3.5%)	33 (4.4%)
Other/Refused	17 (3.6%)	11 (3.9%)	28 (3.7%)
Area of Residence			
Mandalay Region	174 (37.3%)	76 (26.9%)	250 (33.3%)
Yangon Region	124 (26.6%)	77 (27.2%)	201 (26.8%)
Kachin State	92 (19.7%)	33 (11.7%)	125 (16.7%)
Shan State	58 (12.4%)	67 (23.7%)	125 (16.7%)
Tanintharyi Region	19 (4.1%)	30 (10.6%)	49 (6.5%)
Ethnicity			
Bamar	209 (44.8%)	70 (24.7%)	279 (37.2%)
Kachin	90 (19.3%)	32 (11.3%)	122 (16.3%)
Pa’O	49 (10.5%)	47 (16.6%)	96 (12.8%)
Karen	29 (6.2%)	35 (12.4%)	64 (8.5%)
Palaung	28 (6.0%)	29 (10.2%)	57 (7.6%)
Other*	62 (13.3%)	70 (24.7%)	132 (17.6%)
Religion			
Buddhist	331 (70.9%)	198 (70.0%)	529 (70.5%)
Christian/Catholic	132 (28.3%)	84 (29.7%)	216 (28.8%)
Hindu	2 (0.4%)	0 (0.0%)	4 (0.3%)
Muslim	2 (0.4%)	1 (0.4%)	3 (0.4%)

*includes ethnic groups < 25 participants: Chin, Danu, Dawei, Hindu, Intha, Kayah, Kayan, Khmer, Lahu, Lisu, Mon, Mro, Muslim, Rakhine, Shan, Taungyo and mixed ethnicities

### Prevalence and types of conflict-associated stressors

Of the 712 participants who responded to conflict question set, 81.9% (*n* = 583) had experienced at least one conflict-related stressor since the coup (Table 2). The most commonly reported stressors were disruption to participants’ usual education (59.8%) and moving schools as a result of the conflict (33.6%). A substantial portion of participants reported direct exposure to armed combat, such as shelling, shooting or bomb explosions (40.2%), and one-third (33.4%) had been separated from parents or caregivers at some point. Nearly 10% of participants (9.1%) reported losing a family member, and 3.2% said they were injured as a result of the conflict. We found that moving schools and being separated from parents or caregivers due to conflict were more often reported by the older adolescent age group than the younger age group (X^2^ = 5.59, *p* = 0.018 for moving schools; X^2^ = 17.82, *p* < 0.001 for separation).

**Table 2:** Types of conflict-related stressors reported by the participants (*n* = 712)

Question	Early Adolescents: 10–14 years (*n* = 435)	Mid-Late Adolescents: 15–21 years (*n* = 277)	Total (*n* = 712)	X^2^ for early vs. mid/late adolescents
Have you had to flee your home or move house because of the conflict, even if only for one night?	99 (22.8%)	74 (26.7%)	173 (24.3%)	1.23 (*p* = 0.267)
Have you had to move schools due to the conflict?	131 (30.1%)	108 (39.0%)	239 (33.6%)	5.59 (*p* = 0.018)*
Have you been prevented from having access to your usual education due to the conflict?	253 (58.2%)	173 (62.5%)	426 (59.8%)	1.13 (*p* = 0.289)
Have you been separated from your parents/caregivers due to conflict?	119 (27.4%)	119 (43.0%)	238 (33.4%)	17.82 (*p* < 0.001)**
Have you lost any family members due to the conflict?	45 (10.3%)	20 (7.2%)	65 (9.1%)	1.63 (*p* = 0.201)
Have you been directly exposed to armed combat (such as shelling, shooting, bomb explosion)?	168 (38.6%)	118 (42.6%)	286 (40.2%)	0.96 (*p* = 0.328)
Have you suffered any injury as a result of the conflict?	12 (2.8%)	11 (4.0%)	23 (3.2%)	0.46 (*p* = 0.500)

Significance: *p < 0.05, **p < 0.001

### Prevalence of depressive symptoms

Of the 731 participants who answered mental health questions, 94.4% reported at least one symptom of depression, see Table 3. Importantly, 16.1% of respondents reported all five symptoms of depression, and over one quarter (28.2%) reported having considered harming themselves. Across all symptoms among the early and mid-late adolescent age groups, there were no significant differences in depressive symptoms, or the mean depressive symptom count. The mean depressive symptom count was, however, significantly higher among those who lived away from their parents (3.30) compared to those who lived with parents (2.97) using a t-test (*p* = 0.002).

**Table 3:** Depressive symptoms reported by the participants (*n* = 731)

	Early Adolescents: 10–14 years (*n* = 458)	Mid-Late Adolescents: 15–21 years (*n* = 273)	Total (*n* = 731)	X^2^ for early vs. mid/late adolescents
Agree with				
I blame myself when things go wrong	326 (71.2%)	204 (74.7%)	530 (72.5%)	0.91 (*p* = 0.341)
I worry for no good reason	322 (70.3%)	209 (76.6%)	531 (72.6%)	3.06 (*p* = 0.080)
I am so unhappy; I cannot sleep at night	299 (65.3%)	197 (72.2%)	496 (67.9%)	3.40 (*p* = 0.065)
I feel sad	262 (57.2%)	162 (59.3%)	424 (58.0%)	0.24 (*p* = 0.625)
I am so unhappy; I think of harming myself	130 (28.4%)	76 (27.8%)	206 (28.2%)	0.01 (*p* = 0.941)
Depressive symptom count				
Mean depressive symptom count	3.07	3.26	3.14	t-test (*p* = 0.081)

### Association between reports of conflict-related stressors and mental health

**Fig. 1:**
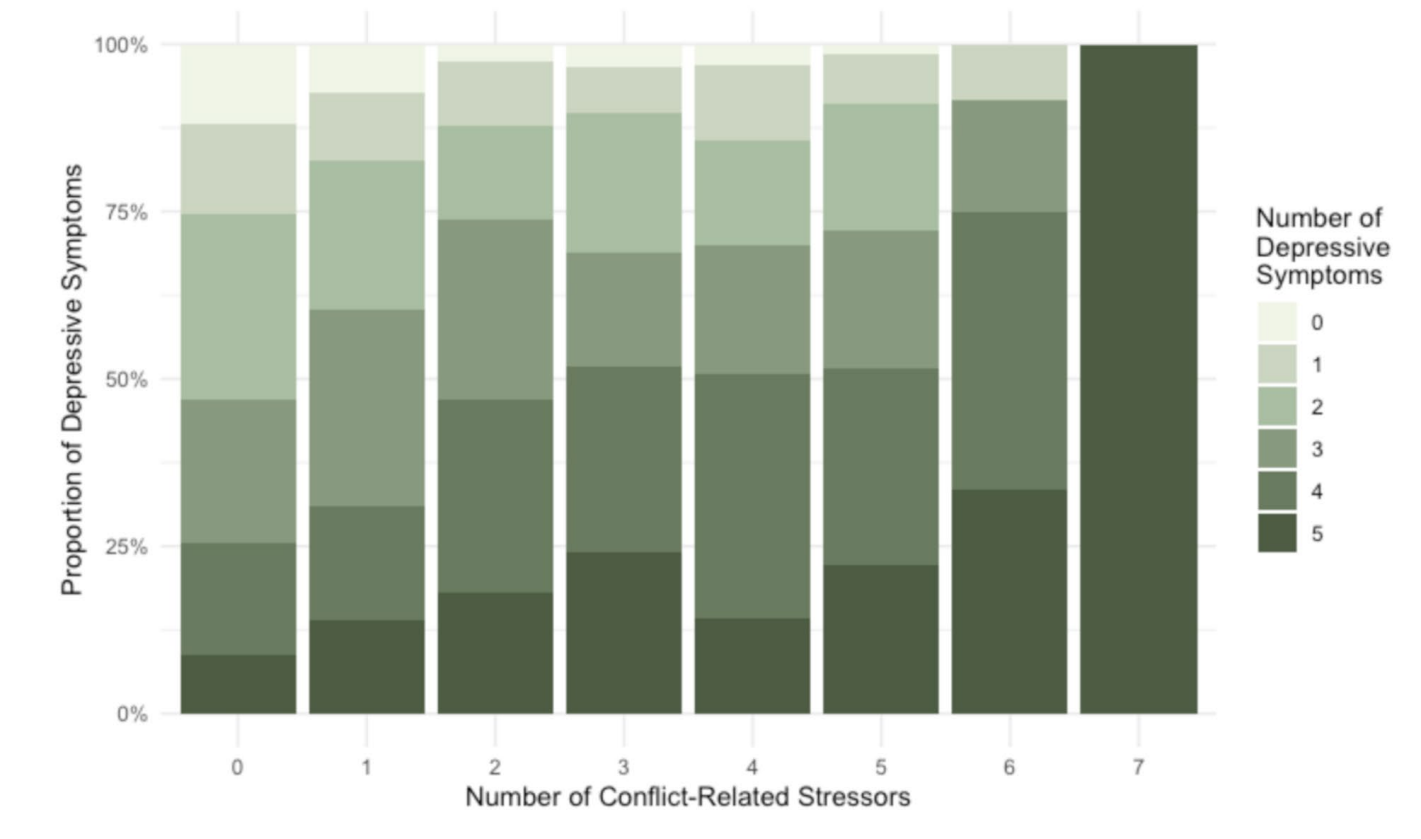
Proportion of depressive symptoms reported by the respondents across different numbers of conflict-related stressors, measured by the reported number of conflict-related stressors by each participant (*n* = 697)

Figure 1 shows the proportion of depressive symptoms compared to the number of conflict-related stressors reported by the respondents, with a deepening colour gradient indicating an increasing number of depressive symptoms. Figure 1 indicates that respondents reporting exposure to more conflict-related stressors also generally reported higher numbers of depressive symptoms, suggesting a positive association between increased exposure to conflict-related stressors and depressive symptom severity. The strength and direction of the association between number of conflict-related stressors and depressive symptoms showed a positive significant association between number of conflict-related stressors and number of depressive symptoms (Rho = 0.231, *p* < 0.001).

We then conducted an ordinal logistic regression to further examine the association between the reported number of depressive symptoms and exposure to the conflict-related stressors. For this analysis, we grouped participants into three groups: those reporting none of the seven conflict-related stressor included in our question set, those reporting one or two conflict-related stressors, and those reporting three or more. The results are presented in Table 4. Results indicate that reporting more conflict-related stressors was significantly associated with reporting more depressive symptoms, even after adjusting for age and whether the respondents lived with their parents. For those reporting one or two conflict-associated stressors, their chances of reporting more depressive symptoms were almost twice as high compared to those who experienced no conflict at all (aOR: 1.97; 95% CI: 1.37, 2.84; *p* < 0.001). For those who reported three or more conflict-associated stressors, their likelihood of reporting more depressive symptoms was almost three times higher than those who reported no conflict-related stressors (aOR: 2.80; 95% CI: 1.86, 4.19, *p* < 0.001).

**Table 4:** Adjusted and unadjusted ordinal logistic regression for the association between exposure to conflict-related stressors and depressive symptoms (*n* = 697)

Conflict level	Reference	OR	95% CI	*p*-value	aOR^1^	95% CI	*p*-value
1–2 stressors (*n* = 342)	No stressors (*n* = 125)	1.97	1.37–2.84	< 0.001**	1.97	1.37–2.84	< 0.001**
3 + stressors (*n* = 230)	No stressors (*n* = 125)	2.79	1.86–4.19	< 0.001**	2.80	1.86–4.19	< 0.001**

^1^Adjusted for age and whether respondents live with their birth parents*p < 0.05, **p < 0.001

When analysing effect modifiers of depressive symptoms using multiple logistic regression, neither being in early or the mid-late age group or living with parents (versus away from parents) changed depression symptoms. However, when stratified by age group (Table 5), our analysis indicates that the number of conflict-related stressors remained a significant predictor of depressive symptoms across age groups. For early adolescents, experiencing any number of conflict stressors remained significantly associated with reporting more depressive symptoms, whereas for mid-late adolescents, only reporting three or more stressors remained significantly associated with more depressive symptoms (aOR: 2.35; 95% CI: 1.24, 4.48; *p* = 0.009). These results suggest that older adolescents’ mental health was only significantly affected by experiencing a higher number of conflict-related stressors, whereas younger adolescents were significantly impacted by any number of conflict-related stressors.

**Table 5:** Adjusted ordinal logistic regression for the association between exposure to conflict-related stressors and depressive symptoms, stratified by age group (*n* = 697)

Early Adolescent age groups (*n* = 429)				
Conflict level	Reference	aOR^1^	95% CI	p-value
1–2 stressors (*n* = 228)	No stressors (*n* = 80)	2.61	1.66–4.12	< 0.001**
3 + stressors (*n* = 121)	No stressors (*n* = 80)	2.98	1.76–45.03	< 0.001**
Mid-Late Adolescent age groups (*n* = 268)				
Conflict level	Reference	aOR^1^	95% CI	p-value
1–2 stressors (*n* = 114)	No stressors (*n* = 45)	1.17	0.63–2.19	0.613
3 + stressors (*n* = 109)	No stressors (*n* = 45)	2.35	1.24–4.48	0.009*

^1^Adjusted for living with birth parents; *p < 0.05; **p < 0.001

When stratified by whether participants were living with their birth parents (Table 6), exposure to three or more conflict-related stressors remained a significant predictor of depressive symptoms across both living situations. However, for participants living away from their parents, reporting one or two stressors was not significantly associated with a higher number of depressive symptoms, meaning only reports of three or more conflict-related stressors was associated with reporting more depressive symptoms.

**Table 6:** Adjusted ordinal logistic regression for the association between exposure to conflict-related stressors and depressive symptoms, stratified by living situation (*n* = 697)

Living with parents (*n* = 340)				
Conflict level	Reference	aOR^1^	95% CI	p-value
1–2 stressors (*n* = 200)	No stressors (*n* = 76)	2.26	1.40–3.62	< 0.001**
3 + stressors (*n* = 64)	No stressors (*n* = 76)	3.00	1.64–5.46	< 0.001**
Living away from parents (*n* = 357)				
Conflict level	Reference	aOR^1^	95% CI	p-value
1–2 stressors (*n* = 142)	No stressors (*n* = 49)	1.58	0.89–2.82	0.118
3 + stressors (*n* = 166)	No stressors (*n* = 49)	2.43	1.37–4.32	0.003*

^1^Adjusted for age; *p < 0.05; **p < 0.001

Ordinal logistic regression analysis for the association between each individual conflict-related exposure and mental health (Table 7) indicates that several conflict-related stressors were significantly associated with increased odds of higher depressive symptom counts. Specifically, being separated from parents/caregivers as a result of the conflict (aOR = 1.77, 95% CI: 1.29–2.42, *p* < 0.001), experiencing a family death (aOR = 2.14, 95% CI: 1.33–3.45, *p* = 0.002), and direct exposure to armed combat (aOR = 2.24, 95% CI: 1.69–2.97, *p* < 0.001) were strongly associated with higher odds of more depressive symptoms. Additionally, fleeing home due to conflict (aOR = 1.50, 95% CI: 1.10–2.04, *p* = 0.011) and being prevent access to usual education (aOR = 1.32, 95% CI: 1.01–1.73, *p* = 0.043) also showed a significant association. No significant associations were found for conflict-associated education disruption, moving schools or suffering any injury.

**Table 7:** Adjusted ordinal logistic regression for the association between reports of the specific conflict-related stressors and depressive symptoms (*n* = 697)

Conflict-related stressors	*n* = “yes”	aOR^1^	95% CI	*p*-value
Have you had to flee your home or move house because of the conflict, even if only for one night?	170	1.50	1.10–2.04	0.011*
Have you had to move schools due to the conflict?	234	1.10	0.83–1.46	0.516
Have you been prevented from having access to your usual education due to the conflict?	419	1.32	1.01–1.73	0.042*
Have you been separated from your parents/caregivers due to conflict?	233	1.77	1.29–2.42	< 0.001**
Have you lost any family members due to the conflict?	62	2.14	1.33–3.45	0.002*
Have you been directly exposed to armed combat (such as shelling, shooting, bomb explosion)?	279	2.24	1.69–2.97	< 0.001**
Have you suffered any injury as a result of the conflict?	21	2.18	0.96–4.99	0.064

^1^Adjusted for age and whether participants lived with their birth parents; *p < 0.05; **p < 0.001

## Discussion

This study sought to understand how adolescent girls’ mental health has been affected by exposure to conflict-related stressors since the 2021 military coup in Myanmar. The first and most critical finding from this study is the direct toll the conflict has taken on young women and girls, with just under half reporting direct experience of armed combat (40.2%), one-third (33.4%) who were separated from their family as a result of the conflict and 10% who had lost family members as a result. Not surprisingly, nearly all participants (94.4%) reported at least one depression symptom: in particular, almost one third of the participants reported that they were so unhappy that they considered harming themselves, and almost 70% reported that they feel so unhappy they cannot sleep at night. Although we did not use a diagnostic tool for depression, the high prevalence of reported depressive symptoms appears comparatively severe compared to similar studies [36], where prevalence rates of mental health symptoms in conflict-affected youth range from 22.2% among Afghan youth [33] to 97% among former child soldiers in Northern Uganda [37]. Furthermore, a systematic review and meta-analysis examining suicidal ideation among women and girls in Southeast Asia reported a prevalence of 16% among adolescent girls, considerably lower than the 28.2% found in our conflict-affected population, suggesting that exposure to armed conflict may substantially increase thoughts of harm [38]. Notably, Mazumder et al.’s review excluded studies involving populations exposed to war.

Our study found that exposure to conflict-related stressors such as displacement, armed combat, family death and disrupted access to education was significantly associated with reporting a higher number of depressive symptoms. These results are in keeping with a recent systematic review on the effects of exposure to armed conflict on mental health, which includes a sub-analysis for youth [39], and support more recent findings from Ukraine [40] and the Gaza Strip [41]. Our study’s finding that 80% of girls experienced at least one conflict exposure, and the significant association with worse mental health, underscores the need for practical strategies that can mitigate the harm to girls’ psychological wellbeing. This is especially pertinent for adolescents, who are mature enough to understand the severity and long term-implications of conflict, and yet still young enough that they are at heightened risk of internalising problems, including post-traumatic stress disorder, anxiety and emotional dysregulation [42].

Our findings indicate that living away from parents could be a substantial risk factor for poor mental health. This finding supports well-established research with conflict-affected youth, for example, Derluyn et al.’s [43] study with refugee adolescents living in Belgium found that being separated from parents, experiencing more traumatising events, and being female were all risk factors for developing serious mental health problems. These results can be understood through the lens of social safety theory, which emphasises the psychological importance of social connectedness and feeling secure within personal relationships [44]. In the context of family separation, the disruption of these core social bonds may activate the brain’s threat response, increasing the likelihood of emotional dysregulation and depressive symptoms [45]. The adolescent girls in our study population who live away from their parents may be experiencing a loss of parental attachment, and therefore a loss of emotional security, which—according to our data—is impacting their mental wellbeing.

Given the strength of the association between living away from parents and poorer mental health and established findings in the broader literature, there is an urgent need to prioritise targeted interventions that address the psychological consequences of living apart from caregivers during conflict. Interventions to enhance a sense of social safety among adolescent girls who are separated from their families, such as those that foster peer- or community-support systems, could be critical to mitigate depressive symptoms in this study population. For example, a review of community-based mental health interventions for conflict affected populations found that community-based interventions were generally effective in improving severe mental health outcomes, including depression [46]. However, few community-based interventions have been robustly studied in conflict-affected populations, and those that have are mostly among refugee populations who have fled conflict, a vast gap in our knowledge remains concerning interventions for those who remain situated in settings of ongoing conflict [46, 47]. In contexts where children are living away from their parents, practical intervention strategies could include the establishment of mentorship programmes and structured peer-support groups within the boarding houses or nunneries [48]. Such interventions could help to rebuild a sense of emotional security, trust and belonging, helping to protect against the psychological effects of conflict and family separation. The association between family separation and worse mental health is notable because across Myanmar, many adolescents and young people live away from their parents so they can access education and avoid dangerous conflict in their hometowns.

Yet, while living with parents may have some protective value, a significant association between conflict exposure and depressive symptoms persisted regardless of living situation. In fact, among respondents living away from parents, reporting one to two conflict-related stressors was no longer significantly associated with more depressive symptoms; only exposure to three or more stressors remained significant. One explanation for this might be that those living away from parents are likely to be from villages or townships where conflict is more severe, and therefore these adolescents may have experienced conflict in the past, and perhaps they have built resilience to lower levels of conflict-related stressors. Additionally, adolescents in boarding schools may have stronger peer social support networks, which have been shown to protect the mental health of displaced youth [49, 50].

A similar effect was found when we stratified by age group: among mid-to-late adolescents, only exposure to three or more conflict-related stressors remained significantly positively associated with depressive symptoms. This may reflect that fact that older adolescents in Myanmar have endured conflicts in the past, particularly adolescents from non-Burmese ethnic nationality groups. Cultural trauma theory would posit that, even adolescents without direct conflict exposure may develop greater awareness of Myanmar’s political history, enabling them to psychologically adapt to “normalise” and contextualise low levels of conflict [51]. Older adolescents may have also formed stronger social support networks, which may weaken the association between conflict exposure and mental health [44]. Still, the data suggest that age is only marginally protective, and exposure to three or more conflict-related stressors significantly impacts mental health, regardless of age or living situation.

Regarding which types of conflict-related stressors were having the most severe impact on mental health, we identified that having the flee their homes, family deaths, prevention of access to usual education, direct exposure to armed combat, family separation due to conflict were having the most profound effects on the participants mental health. These findings offer insights into the specific types of conflict-related exposures that may be the most useful targets for intervention, specifically when prioritising support to the most severely impacted groups. Our results suggest that interventions should target girls separated from their parents, those who have been displaced, those out of school and those who are in the closest physical proximity to armed combat.

The significant association between conflict-related stressors and depressive symptoms underscores the profound psychological impact of conflict on adolescents and emphasises the need for tailored mental health interventions for conflict-affected youth. Mental health and psychosocial support have historically been overlooked in emergency settings, despite crises and their aftermaths often being characterised by a disproportionately high burden of mental disorders [4]. Furthermore, when considering adolescent girls’ mental health during times of conflict, substantial research has focused on risk factors for negative mental health outcomes, but there has been less research on protective factors to promote positive mental health, such as building social support structures or maintaining hope [52, 53]. A greater understanding of protective factors is required if we are to develop holistic interventions to protect the mental health of young people during conflict [12]. One clear positive finding of this research is that 97.5% of our study population were enrolled in some form of education. Although this may be an artefact of our sampling strategy, it suggests that school-based interventions to support mental health are a viable and potentially scalable option for this study population. In Myanmar, where humanitarian access and resources are highly constrained, practical strategies could include training teachers and boarding house staff in basic psychosocial support, developing peer-support groups and embedding promotion of mental health into existing extracurricular programming.

There are important limitations when interpreting the results of this study. First, although this study focuses on adolescent girls, future research should also explore the experiences of boys, young men, and gender non-conforming adolescents in conflict settings, in order to inform inclusive and comprehensive mental health interventions. Next, there may be residual confounding, which can occur when there are inadequately measured variables that influence both the exposure and the outcome but were not included in our analysis [54]. For example, factors such as socioeconomic status, pre-existing mental health conditions, or specific reasons for moving away from their parents were not accounted for and therefore may partially explain the observed relationships. Furthermore, due to the cross-sectional nature of this study, we cannot ascertain causality between conflict-related stressors and depressive symptoms, as the depressive symptoms may have preceded recent conflict. Also, due to the limited time and resources, we were unable to use validated tools which are particularly important when trying to measure subjective evaluations of mental health. Still, the survey tool was rigorously piloted and co-developed with a team of peer-researchers to increase the content and construct validity.

While our stratified analyses by living arrangement and age group are informative and feasible, certain subgroups had relatively modest sample sizes (e.g., *n* = 45–64), which limits statistical power and estimate stability. Therefore, the stratified results should be interpreted with caution. Our finding that one to two conflict-related stressors were not significantly associated with higher depressive symptoms among participants living away from their parents/caregivers should also be interpreted with caution. As being separated from parents was both a conflict-related stressor and a defining variable within this subgroup, participants in the 1–2 stressors category may have reported only one additional exposure beyond parental separation, potentially limiting variation in exposure count within this subgroup. This may have reduced the power to detect statistically significant associations, despite a consistent trend in odds ratios. Finally, although we tried our best to conduct our survey with a random sample, the circumstances in Myanmar meant that it was not always possible to ensure this, as often survey sites had to be changed last minute due to safety and accessibility concerns. Our sample consisted of girls who were currently enrolled in GD’s empowerment programme, and as such our findings may not be representative of girls outside the programme. Our study also used region-based sampling, which may cause clustering effects by region. Due to the limited number of regions and modest sample sizes per region, we did not incorporate multilevel modelling or clustering adjustments and therefore we have not explicitly accounted for potential clustering at the regional level. Future research with larger sample sizes could further investigate and address these potential clustering effects.

To ensure the safety of our research team and study participants, this study was conducted in regions of Myanmar where the conflict was less severe at the time of the survey. This means that the concerning results presented here are likely an underrepresentation of the severity of the conflict situation in Myanmar and therefore will not be capturing the full scale of the mental health crisis in the country. The devastating earthquake that hit Myanmar at the end of March 2025, adds an additional layer of hardship to an already deeply disadvantaged population. This disaster will undoubtedly exacerbate existing conflict-related adversities, with significant and far-reaching implications for girls’ education, mental health, physical safety, and aspirations for the future.

## Conclusions

Given the growing number of countries experiencing unrest, war and displacement, there is an urgent need for more research into interim interventions to safeguard young people’s mental health in situations of ongoing conflict. Our findings suggest that fostering social safety among adolescent girls who are living away from their families—for example, through peer- or community-support systems—may help to mitigate depressive symptoms. Moreover, the pervasive gender inequalities that severely disadvantage adolescent girls and young women in these settings underscore the importance of developing practical, scalable and protective gender-sensitive interventions. These efforts should aim to not only mitigate the immediate mental health needs related to conflict, but also to prevent long-term repercussions for adolescent girls’ and young women’s future wellbeing.

## Data Availability

Anonymised versions of datasets analysed in this study are available from the corresponding author upon reasonable request.

## Abbreviations

COVID-19: Coronavirus Disease 2019
PTSD: Post-Traumatic Stress Disorder
NGO: Non-Governmental Organisation
GD: Girl Determined
GEAS: Global Early Adolescent Study
CI: Confidence Interval
aOR: Adjusted Odds Ratio
